# Prognostic significance of hsa_circ_0048122 to predict liver metastasis in early‐stage colorectal cancer

**DOI:** 10.1002/jcla.24577

**Published:** 2022-07-06

**Authors:** Qian Fang, Chuandou Ni, Zhun Cai, Wangyong Li, Jianjin Xie

**Affiliations:** ^1^ General Surgery, The First People's Hospital of Wenling Wenling China; ^2^ Anorectal Surgery, The First People's Hospital of Wenling Wenling China

**Keywords:** biomarker, colorectal cancer, hsa_circ_0048122, liver metastasis, therapy

## Abstract

**Background:**

Liver metastasis is the primary cause of lethal colorectal cancer (CRC). The predominant risk of poor patient prognosis in early‐stage CRC emerges as metachronous liver metastasis. This necessitates the search for potential biomarkers for this metastasis to assess treatment outcomes and provide targeted therapy.

**Methods:**

The role of hsa_circ_0048122 in predicting liver metastasis in CRC was probed in this work. This retrospective and multi‐center investigation entailed exploration and identification stages with 158 and 176 patients. While RT‐qPCR was employed to scrutinize hsa_circ_0048122 expression, Kaplan–Meier survival, and multivariate analyses were used to probe its prognostic impact in early‐stage CRC and stage IV CRC cases, respectively.

**Results:**

A strong correlation between liver metastases and hsa_circ_0048122 expression in stage IV CRC patients with a high hsa_circ_0048122 profile indicated a poor overall survival. Likewise, a high expression level of hsa_circ_0048122 appears as a potential predictor of liver metastases in patients' initial stages.

**Conclusions:**

Predicting liver metastasis can be plausibly facilitated using Hsa_circ_0048122 as a biomarker in early‐stage CRC cases.

## Graphical abstract

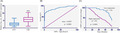

## 1 INTRODUCTION

Colorectal cancer (CRC) is one of the most common cancers, and it is the third primary cause of cancer‐related deaths worldwide.^1^, ^2^, ^3^ The vital involvement of metastasis in this mortality is known. This metastatic colonization site's primary location is the liver because of hepatic artery and portal drainage.^4^ Synchronous liver metastases (CRCLM) account for close to one‐fifth of CRC diagnoses, with the 5‐year overall survival (OS) lower than 15% in such patients.^1^ Even though when this diagnosis is made in early‐stage (TNM stage I/II), the 5‐year OS can be as high as 90%. The post‐operative metachronous liver metastasis recorded in a few patients can impact the clinical outcomes. The 5‐year cumulative occurrence of this metastasis is 3.7% and 13.3% in TNM stage II and stage I CRC malignancies, respectively.^5^ The random occurrence of metachronous metastasis among patient groups is most likely responsible for the molecular heterogeneity in CRC. As a new research avenue, this necessitates the investigation of novel biomarkers for assessing and predicting this risk.

Circular RNAs (circRNAs) are vitally functioning in transcriptional and posttranscriptional control of gene expression and have covalently closed‐loop structures without a 5 “cap or a 3” poly A tail.^6^, ^7^, ^8^ Despite their discovery more than four decades ago, they have recently attracted significant interest. High‐throughput sequencing has facilitated the identification of more than 30,000 circRNAs. While exonuclease targets their parental linear RNAs, circRNAs are resistant because of their loop structure. This stability accounts for circRNA abundance in mammalian cells. Their occurrence in specific pathologies and stages^9^ is indicative of their possible role as novel markers for therapy and diagnosis.^10^ The close association between oncogenesis and circRNAs is being documented, with their vital functioning in epithelial–mesenchymal transition being reported in studies. This indicates that their putative functioning impacts several aspects of tumorigenesis like invasion, migration, and proliferation.^11^, ^12^, ^13^ The probable involvement of circRNAs in CRC progression is also emerging. For example, the promotion of CRC progression by circRNA_0000392 via the miR‐193a‐5p/PIK3R3/AKT axis was reported.^14^ Other molecules roles along similar lines were exosomal circPACRGL via the miR‐142‐3p/miR‐506‐3p‐TGF‐β1 axis^15^ and hsa_circRNA_002144 through the miR‐615‐5p/LARP1/mTOR pathway.^16^ This led us to discover a new circRNA (hsa_circ_0048122) as a potential biomarker for predicting metachronous liver metastatic risk in CRC.

This study involves the comprehensive investigation of two case groups: (1) the CRCLM group inclusive of subjects with the metastasis of the liver during diagnosis subjected to primary CRC malignancy removal and liver metastases resection and (2) the early‐stage CRC group comprising patients with stage I/II malignancies subjected to R0 resection. Hsa_circ_0048122 emerged as an independent criterion for OS prognosis in the CRCLM group. Elevated hsa_circ_0048122 levels also appear as a credible biomarker of the risk of liver metastasis in the second group.

## 2 METHODS

### 2.1 Patient specimens

CRCLM patient specimens and paired synchronous liver metastases (*n* = 158) were collected. The enrolment of an additional set of CRC patients (stage I/II; *n* = 176) was also done. Patients reporting liver metastasis within 12 months post‐surgery were excluded. The Wenling First People's Hospital provided the data and specimens. Wenling First People's Hospital's Ethics Committee approved this study. All patients signed written informed consent forms. Moreover, the CONSORT Statement's guidelines were followed in this research.

### 2.2 RNA extraction and RT‐qPCR

TRIzol reagent (Invitrogen, Thermo Fisher Scientific,) was employed for sample RNA isolation per the requisite and recommended instructions. Reverse transcription of around 2 μg of total RNA into cDNA ensued with the RTqPCR performed following an earlier protocol.^17^ The internal standard was GAPDH. The primers utilized in this study are listed in Table 1.

**TABLE 1:** Primer sequences used in the present study

Name	Primer sequences (5′‐3′)
hsa_circ_0048122	
Forward	TGCTGACCGTCATCCTGGC
Reverse	ATGACGGTCAGCAGGGGC
GAPDH	
Forward	GCACCGTCAAGGCTGAGAAC
Reverse	ATGGTGGTGAAGACGCCAGT

### 2.3 Statistical analysis

OS was the time from tumor resection to the last follow‐up or death date. The time between tumor resection and diagnosing liver metastases was considered liver metastasis‐free survival (LMFS). Graphpad Prism7 software was utilized for all analyses. While the Chi‐square test was used to score hsa_circ_0048122 expression and clinicopathological parameters correlations, the prognostic values were probed by Kaplan–Meier analysis and Cox regression analysis. Statistical significance was at *p* < 0.05 (*).

## 3 RESULTS

### 3.1 CRCLM patient circRNA profiling

**FIGURE 1:**
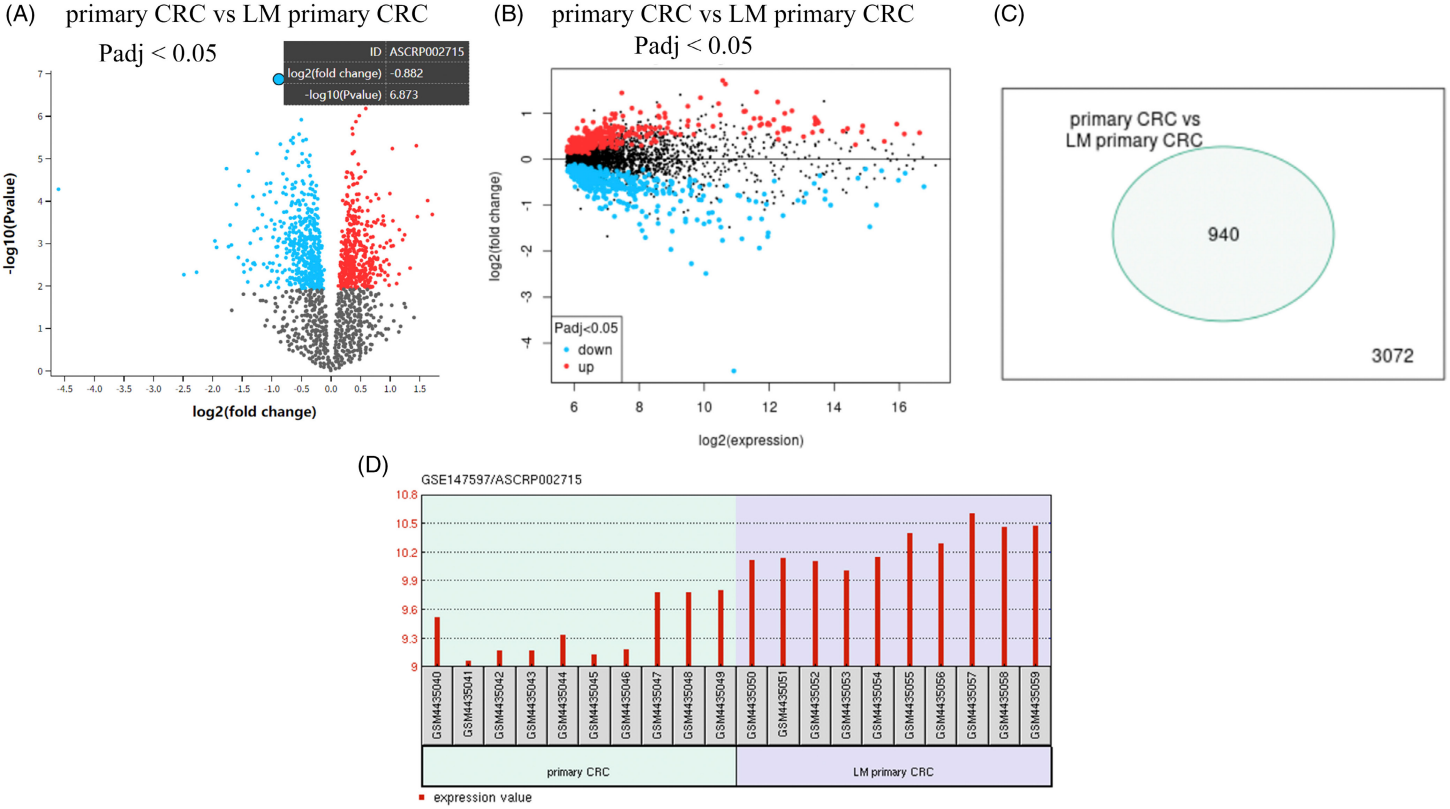
Profiling of circRNAs from the CRCLM patients. (A) Volcano plot of differentially expressed genes in GEO datasets GSE147597. (B) Mean‐difference plot in GEO datasets GSE147597. (C) Venn diagram analysis of differentially expressed genes. (D) Hsa_circ_0048122 is upregulated in NSCLC compared to adjacent normal tissues

The circRNA significantly dysregulated in CRCLM were identified based on the three GEO datasets. Based on the analysis of the cohorts, 940 differentially expressed genes were demonstrated from GSE158695 datasets (Figure 1A,B,C). In addition, we identified all the highly expressed genes that could differentiate CRC with liver metastasis from CRC devoid of liver metastasis in each independent dataset (Figure 1C). Hsa_circ_0048122 analysis revealed the highest expression in CRC with liver metastasis vs CRC without liver metastasis (Figure 1D). As a result, we selected circRNA_001846 for further investigation.

### 3.2 Prognostic value of hsa_circ_0048122 in stage IV patients with CRCLM

**FIGURE 2:**
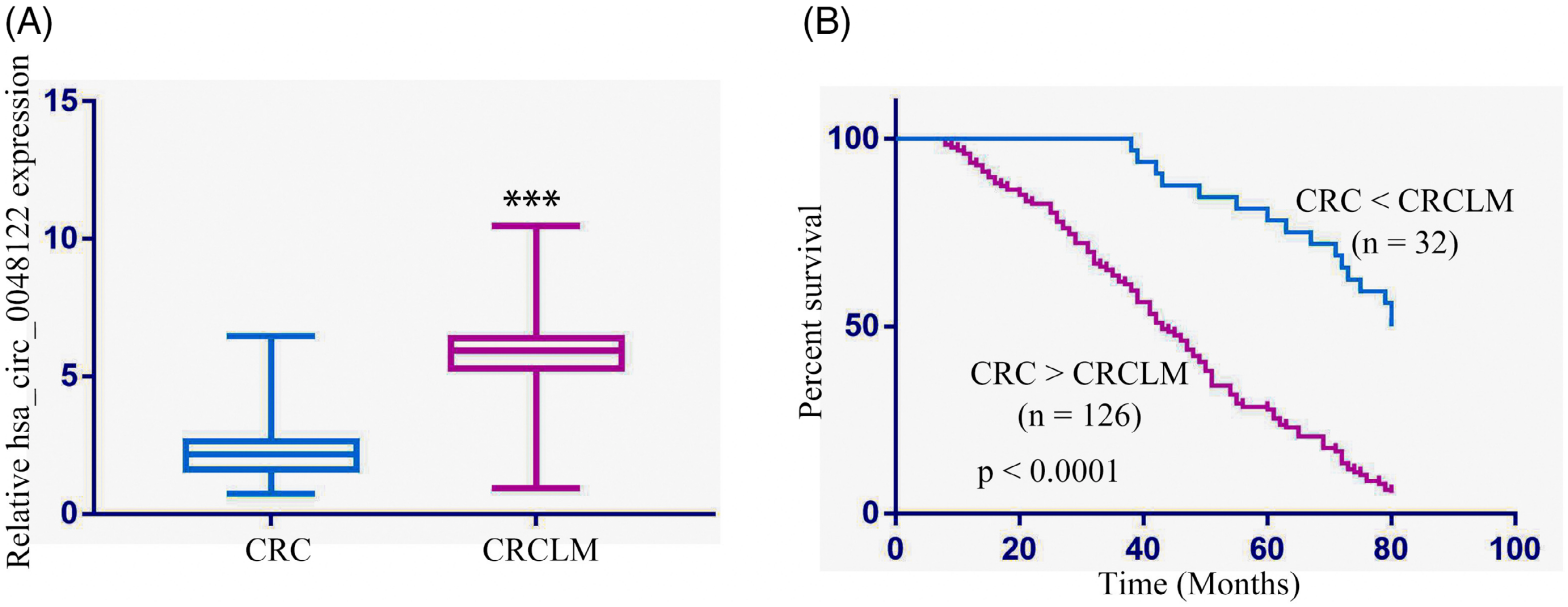
Prognostic value of hsa_circ_0048122 in stage IV patients with CRCLM. (A) Hsa_circ_0048122 in initial CRC tissues and paired liver metastases. (B) Higher expression of hsa_circ_0048122 in CRCLM demonstrates poor overall survival (OS)

Higher hsa_circ_0048122 mRNA levels were detected in CRCLM tissues compared to the primary CRC tissues (Figure 2A). Table 2 displays the clinical characteristics of the stage IV CRCLM patients (*n* = 158). The median age was 63 years (range from 35 to 82 years), with primary colon cancer constituting 84 specimens (53.2%) and primary rectum cancer accounting for the remaining 74 cases (46.8%). Most patients (*n* = 85 or 72.2%) experienced unilobar liver metastases, and 53.2% presented with less than three metastases.

**TABLE 2:** Characteristics of stage IV cases with CRCLM

Variables	hsa_circ_0048122 expression		*p*‐value
	Low	High	
Gender			0.245
Female	36	33	
Male	43	46	
Age (year)			0.315
≤60	29	37	
>60	50	42	
Primary tumor location			0.194
Colon	41	43	
Rectum	38	36	
Primary tumor differentiation			0.537
Poor	37	40	
Well/moderate	42	39	
CRCLM distribution			0.796
Unilobar	55	59	
Bilobar	24	20	
No. of CRCLM			0.022
<3	62	22	
≥3	17	57	
Resection margin			0.271
R0	51	53	
R1	28	26	

The expression of hsa_circ_0048122 in liver metastases vs primary tumor specimens (the CRCLM/CRC ratio) was applied to categorize our cohort into two subgroups. Higher hsa_circ_0048122 expression was detected in CRCLM over CRC samples in 158 cases. A positive correlation was observed between this increased level and the number of liver metastases (*p* = 0.022, Table 2). A correlation also emerged between the high hsa_circ_0048122 expression with poor OS in CRCLM, as showed by Kaplan–Meier survival analyses (*p* = 0.002, Figure 2B, Table 3). The distribution of liver metastases (*p* = 0.024), metastases number (*p* = 0.035), and resection margins (*p* = 0.011) were determined as additional prognostic factors. Hsa_circ_0048122 appears as an independent biomarker for CRCLM prognosis as demonstrated by the multivariate analysis (risk ratio [RR] =3.05, 95% confidence interval [CI] =0.942–7.657, *p* = 0.019, Table 4). The other independent prognostic factors were the distribution of liver metastases and resection margins.

**TABLE 3:** Overall survival (OS) of stage IV cases with CRCLM

Variables	Cases (*n* = 158)	3‐year OS (%)	Mean OS time (months)	*p*‐value
Gender				1.025
Female	69	34.8	33.2 ± 4.9	
Male	89	42.7	34.5 ± 3.7	
Age (year)				0.945
≤60	66	47.0	34.5 ± 5.9	
>60	92	33.7	34.3 ± 4.2	
Primary tumor location				0.342
Colon	84	46.4	37.5 ± 6.4	
Rectum	74	31.0.1	29.4 ± 5.7	
Primary tumor differentiation				0.127
Poor	77	45.5	43.5 ± 5.9	
Well/moderate	81	33.3	30.2 ± 4.1	
CRCLM distribution				0.024
Unilobar	114	45.6	41.5 ± 4.6	
Bilobar	44	22.7	19.5 ± 3.7	
No. of CRCLM				0.035
<3	84	58.3	40.4 ± 3.6	
≥3	74	17.6	18.3 ± 5.4	
Resection margin				0.011
R0	104	53.8	37.4 ± 4.5	
R1	54	11.1	13.3 ± 3.8	
hsa_circ_0048122 expression				<0.0001
CRCLM ≤ CRC	32	81.3	55.6 ± 4.8	
CRCLM > CRC	126	28.6	26.5 ± 3.7	

**TABLE 4:** Cox regression analysis of stage IV cases with CRCLM

Variable	HR	95% CI	*p*‐value
CRCLM distribution (bilobar vs unilobar)	2.56	1.63–6.24	0.034
No. of CRCLM (≥3 vs <3)	1.73	0.552–2.943	0.652
Resection margin (R1 vs R0)	3.96	1.542–12.49	0.024
hsa_circ_0048122 expression pattern (CRCLM > CRC vs CRCLM ≤ CRC)	3.05	0.942–7.657	0.019

### 3.3 Correlation between hsa_circ_0048122 expression and liver metastasis in early‐stage CRC specimens

**FIGURE 3:**
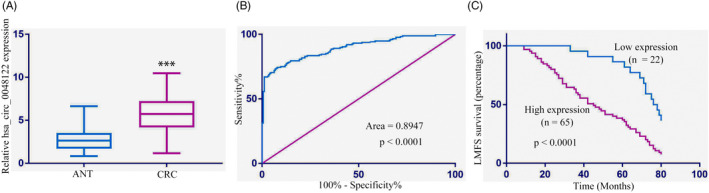
Hsa_circ_0048122 expression is associated with liver metastasis in early‐stage CRC cases. (A) Hsa_circ_0048122 in early‐stage CRC tissues and paired adjoining non‐tumorous tissues (ANT). (B) Hsa_circ_0048122’s ROC curves suggested that it could be a worthy marker for predicting CRC cases. (C) High levels of hsa_circ_0048122 expression were associated with poor liver metastasis‐free survival (LMFS)

The aforementioned_circ_0048122 profile and its impact on CRCLM patients (stage IV) led us to investigate its effects in predicting the metachronous liver metastasis risk in initial grade specimens. Therefore, has_circ_0048122 was tested in early‐stage CRC tumors and non‐malignant samples for this study. The RT‐qPCR experiment revealed an increase of hsa_circ_0048122 in tumor tissues (Figure 3A), showing that hsa_circ_0048122 is oncogenic in CRC. The ROC curves suggested that it could be a credible marker for predicting metastasis in CRC patients (Figure 3B).

As a result, we included another retrospective cohort of stage I/II CRC patients (*n* = 176) as the identification group. A correlation emerged between increased hsa_circ_0048122 expression and bigger tumor size (*p* = 0.022) and liver metastasis (*p* = 0.009) (Table 5). It raised the possibility of the has_circ_0048122 being involved in distant CRC metastases. The testing of our hypothesis entailed the selection of 87 cases (49.4%) from the 176 samples with metachronous liver metastasis and a subsequent Kaplan–Meier survival analysis, validating the correlation of increased hsa_circ_0048122 expression and high liver metastasis risk (LMFS: 49.3 ± 5.2 vs 73.5 ± 7.1 months, *p* = 0.005. Figure 3C, Table 6). Overall, our findings show that hsa_circ_0048122 has the potential as a predictor of liver metachronous metastasis in early‐stage CRC. This opens the door to a more in‐depth examination of clinical outcomes and targeted therapy.

**TABLE 5:** Characteristics of early‐stage CRC patients

Variables	hsa_circ_0048122 expression		*p*‐value
	Low	High	
Gender			0.654
Female	41	42	
Male	47	46	
Age (year)			0.325
≤60	44	46	
>60	44	42	
Primary tumor location			0.257
Colon	55	51	
Rectum	33	37	
Primary tumor size			0.022
<5 cm	61	23	
≥5 cm	27	65	
Primary tumor differentiation			0.672
Poor	26	19	
Well/moderate	62	69	
TNM stage			0.943
I	29	27	
II	59	61	
Liver metastasis			0.009
Absent	66	23	
Present	22	65	

**TABLE 6:** Liver metastasis‐free survival (LMFS) of early‐stage CRC cases

Variables	Cases (*n* = 87)	5‐year LMFS (%)	Mean LMFS (months)	*p*‐value
Gender				0.642
Female	36	47.2	58.1 ± 5.7	
Male	51	47.1	57.6 ± 3.9	
Age (year)				0.519
≤60	44	52.3	59.2 ± 5.9	
>60	43	41.9	51.4 ± 5.5	
Primary tumor location				0.559
Colon	75	45.3	57.5 ± 5.1	
Rectum	12	58.3	51.7 ± 6.2	
Primary tumor differentiation				0.627
Poor	21	66.7	51.3 ± 4.2	
Well/moderate	66	40.9	57.6 ± 5.2	
Primary tumor size				0.067
<5 cm	21	57.1	72.5 ± 3.7	
≥5 cm	66	43.9	50.3 ± 4.5	
TNM stage				0.492
I	15	53.3	51.6 ± 12.5	
II	72	45.8	55.1 ± 5.1	
hsa_circ_0048122 expression				<0.0001
Low	22	86.4	73.5 ± 7.1	
High	65	33.8	49.3 ± 5.2	

## 4 DISCUSSION

The most deadly cause of CRC is liver metastasis.^18^ Despite the primary treatment strategy of surgical resection in CRCLM patients, the 5‐year OS is less than 15%, decreasing the clinical outcome. At an initial stage, the CRC diagnosis of metachronous distant metastasis in the liver is primarily linked to an unfavorable prognosis. The wide range of metachronous liver metastasis patterns observed in individuals justifies the search for new biomarkers to help anticipate the disease and improve prognosis.

The transformation of original tumor cells in a sequential metastatic cascade system is required for CRC malignant cell invasion and subsequent metastasis.^19^ Metastasis research casts the limelight on circRNAs as target candidates for therapy.^20^, ^21^, ^22^ The vital involvement of circRNAs in CRC metastasis is being documented. An example is promoting CRC tumorigenesis by circ‐ERBIN via miR‐125a‐5p and miR‐138‐5p/4EBP‐1 mediated cap‐independent HIF‐1α translation.^23^ CRC proliferation and metastasis are promoted by hsa_circ_0006401,^24^ while circ_0026344 checks CRC metastasis via miR‐183.^25^ Notably, circRNAs are excellent biomarkers. For instance, a novel potential biomarker for diagnosing breast cancer was serum Circ‐FAF1/Circ‐ELP3 in one report.^26^ Similar biomarkers were outlined for gastric cancer using circRNA microarray profiling,^27^ such as hsa_circ_0001020 for screening and prognosis of this malignancy.^28^ Similarly, circRNAs have also been widely used as markers in CRC diagnosis, including hsa_circ_0002320,^29^ hsa_circ_001659,^30^ hsa_circ_0004585,^31^ circ_0026344,^32^ and hsa_circ_0001649.^33^ However, biomarkers for CRCLM diagnosis are not fully comprehended. Our scrutiny of the GEO datasets and stage IV CRCLM patient datasets revealed the hsa_circ_0048122 as a promising candidate. This is based on the rationale given below: 1) the evident correlation between hsa_circ_0048122 expression and the liver metastases number and 2) hsa_circ_0048122 levels appeared as an independent factor for prognosis of CRCLM patient OS. An increased hsa_circ_0048122 expression indicated a worse clinical outcome in liver metastases tissues.

A dataset of early‐stage CRC patients (*n* = 162) was examined as part of an identification cohort. The increased hsa_circ_0048122 levels were associated with post‐surgery metachronous liver metastasis. That suggested that hsa_circ_0048122 may influence the tumorigenic characteristics. Further probing in a validation cohort composed of metachronous liver metastasis samples (*n* = 38) corroborated the potential of hsa_circ_0048122 as a promising biomarker to predict LMFS. This is the first study to show that a high hsa_circ_0048122 profile in CRC patients can potentially indicate metachronous liver metastasis in such cases.

Although has_circ_0048122 is documented in CRCLM, its mechanical characteristics and function require further investigation. This study revealed the previously unknown role of has_circ_0048122 in CRC malignancy. Future investigation and research studies are needed to examine and search for a deeper understanding of the functioning and associated molecules of has_circ_0048122.

## 5 CONCLUSION

In conclusion, the potential role of has_circ_0048122 as an independent factor in stage‐IV CRCLM patient OS prognosis and liver metastasis in early‐stage CRC patients was identified.

## CONFLICT OF INTEREST

The authors declare no conflict of interest.

## Data Availability

Due to the nature of this research, participants of this study did not agree for their data to be shared publicly, so supporting data is not available.
